# Single nucleotide polymorphisms in native South American Atlantic coast populations of smooth shelled mussels: hybridization with invasive European *Mytilus galloprovincialis*

**DOI:** 10.1186/s12711-018-0376-z

**Published:** 2018-02-22

**Authors:** Małgorzata Zbawicka, María I. Trucco, Roman Wenne

**Affiliations:** 1 Institute of Oceanology, Polish Academy of Sciences, Powstańców Warszawy 55, 81-712 Sopot, Poland; 2 Instituto Nacional de Investigación y Desarrollo Pesquero, Paseo Victoria Ocampo No. 1, B7602HSA Mar Del Plata, Buenos Aires Argentina

## Abstract

**Background:**

Throughout the world, harvesting of mussels *Mytilus* spp. is based on the exploitation of natural populations and aquaculture. Aquaculture activities include transfers of spat and live adult mussels between various geographic locations, which may result in large-scale changes in the world distribution of *Mytilus* taxa. *Mytilus* taxa are morphologically similar and difficult to distinguish. In spite of much research on taxonomy, evolution and geographic distribution, the native *Mytilu*s taxa of the Southern Hemisphere are poorly understood. Recently, single nucleotide polymorphisms (SNPs) have been used to clarify the taxonomic status of populations of smooth shelled mussels from the Pacific coast of South America. In this paper, we used a set of SNPs to characterize, for the first time, populations of smooth shelled mussels *Mytilus* from the Atlantic coast of South America.

**Results:**

*Mytilus* spp. samples were collected from eastern South America. Six reference samples from the Northern Hemisphere were used: *Mytilus edulis* from USA and Northern Ireland, *Mytilus trossulus* from Canada, and *Mytilus galloprovincialis* from Spain and Italy. Two other reference samples from the Southern Hemisphere were included: *M. galloprovincialis* from New Zealand and *Mytilus chilensis* from Chile. Fifty-five SNPs were successfully genotyped, of which 51 were polymorphic. Population genetic analyses using the STRUCTURE program revealed the clustering of eight populations from Argentina (*Mytilus platensis*) and the clustering of the sample from Ushuaia with *M. chilensis* from Chile. All individuals in the Puerto Madryn (Argentina) sample were identified as *M. platensis* × *M. galloprovincialis* F2 (88.89%) hybrids, except one that was classified as Mediterranean *M. galloprovincialis*. No F1 hybrids were observed.

**Conclusions:**

We demonstrate that *M. platensis* (or *Mytilus edulis platensis*) and *M. chilensis* are distinct native taxa in South America, which indicates that the evolutionary histories of *Mytilus* taxa along the Atlantic and Pacific coasts differ. *M. platensis* is endangered by hybridization with *M. galloprovincialis* that was introduced from Europe into the Puerto Madryn area in Argentina, presumably by accidental introduction via ship traffic. We confirm the occurrence of a native *M. chilensis* population in southern Argentina on the coast of Patagonia.

**Electronic supplementary material:**

The online version of this article (10.1186/s12711-018-0376-z) contains supplementary material, which is available to authorized users.

## Background

Correct recognition and identification of species are important to understand the phylogeography of living resources and to ensure the conservation of their biodiversity, their management, sustainable exploitation and traceability. The world production of mussels, *Mytilus* spp., including exploitation of natural populations and aquaculture, is approximately 1.2 million tons per year [1, 2]. Aquaculture activities include the transfer of spat and live adult mussels between geographic locations, which may result in large-scale changes in the world distribution of *Mytilus* taxa [3–5]. *Mytilus* taxa are morphologically similar and difficult to distinguish. Replacement of native species by an invasive taxon can go unnoticed [6]. Individuals from different *Mytilus* taxa can hybridize in areas where their populations merge and coexist. Hybridization zones have been described in the Danish Straits at the entrance to the Baltic Sea [7–10], on the Atlantic coasts of France [11], Great Britain [12, 13], Greenland [14], and the Atlantic coast of Canada [15]. In addition, the occurrence of doubly uniparental inheritance of mitochondrial DNA (mtDNA), of recombination and introgression can hinder the use of mtDNA analyses for the identification of *Mytilus* taxa [16–19]. Nuclear DNA markers including genome-wide single nucleotide polymorphisms (SNPs) can indicate geographic origin of interbreeding taxa when hybridization occurs either naturally or results from the introduction of mussels to non-native regions [14, 20].

Introduction of *Mytilus galloprovincialis*, one of the most invasive mussel species, threatens native populations of *Mytilus* on a global scale [21, 22]. Irrespective of the ecological consequences, including reduction of abundance, introductions of this species have usually been followed by hybridization of *M. galloprovincialis* with native *Mytilus* taxa, i.e. *Mytilus trossulus* on the eastern and western North Pacific coasts [23–26] and native Southern Hemisphere *M. galloprovincialis* in Australia and New Zealand [27–29]. Michalek et al. [30] reviewed the negative consequences of such hybridization events for the culture of *Mytilus* in Europe. The introduced *M. galloprovincialis* interbreeds with native *Mytilus edulis* are cultivated in Scotland. Northern Hemisphere *M. galloprovincialis* is also a threat for local farms that are located south of the Gulf of Arauco in Chile and use native populations of *Mytilus chilensis* from South America [31].

The longest Pacific and Atlantic coastlines that are continuously inhabited by native populations of *Mytilus* are in South America. South American smooth shelled blue mussels of the genus *Mytilus* are present on the Atlantic coast of the south of Brazil [32], through Uruguay and Argentina down to Tierra del Fuego [33, 34] and around Cape Horn, and then north into the Pacific to Golfo de Arauco, Chile [35], where they inhabit both intertidal levels and deep banks. The nomenclature for these mussels has been controversial; they were classified based on fossil records and morphological data as *Mytilus edulis* [34], *M. platensis* [33, 36–38] or *M*. *chilensis* [39]. The native species of *Mytilus* on the Atlantic coast of South America, i.e. *M. platensis* was described by d’Orbigny in 1846 [40] using specimens that were collected from the area of Maldonado in Uruguay. The native species on the Pacific coast of South America, i.e. *M. chilensis* was described by Hupé in 1856 [41] based on specimens found near Concepcion in Chile. Argentine mussels, which were first identified as *M. platensis*, were later synonymised to *M. edulis* or assigned a subspecies rank [38, 42]. Ecological studies also created confusion by assigning specific names, i.e. mussels from the south of Brazil, Uruguay and Buenos Aires province were designated *M. platensis* [43, 44] and later as *M. edulis platensis* [45], while those found in Atlantic Patagonian waters and gulfs were variably named, i.e. *M. platensis* [39], *M. chilensis* [46] and even *M. edulis* *chilensis* [47, 48]. Based on morphological characters such as color, hinge teeth, valve thickness and inferior margin, Castellanos [33] named the Argentine mussel *M. platensis* to differentiate it from the Chilean mussel, *M. chilensis*. Later, these mussels were considered as subspecies (*M. edulis platensis* and *M. edulis* *chilensis*) since the number of diagnostic characters to assign them to species rank was not sufficient [32, 49]. For fishery management purposes, the names *M. e. platensis* and *M. chilensis* have been used in Argentina and Chile, respectively.

Aquaculture of Atlantic *M. e. platensis* was developed in Argentina [50]. Currently, suspended commercial cultures operate in Nuevo Gulf (Puerto Madryn), San Jose Gulf (Isla de los Pájaros) and San Matías Gulf (San Antonio Oeste–Puerto Lobos) [51]. Production of cultured mussels (*Mytilus* and *Aulacomya*) in Argentina fell from over 250 tons in 2011 to 11.20 tons in 2016 [52, 53]. Due to economic reasons, *M. e. platensis* is cultivated only in the San Matías Gulf [54] and *M. chilensis* in the Canal Beagle, Ushuaia [55].

The natural distribution of Chilean mussels ranges from Bahia Concepcion (36°45′S) to Punta Arenas (53°S), on the edge of the Strait of Magellan [56, 57]. There have been many studies to clarify their taxonomic status in connection with intensive aquaculture. Various studies using allozymes, nuclear and mitochondrial DNA markers have led to contradictory results, depending on the type of marker used. The presence of *Mytilus edulis* [58, 59] and *M. galloprovincialis* [35, 56, 57, 60, 61] was reported in Chile, although it is not clear if these are native or introduced populations [62], or even different species that may have been included in a single taxon as *M. chilensis* [63]. Based on morphological and molecular analyses, Toro [64] suggested that *M. chilensis* was a subspecies, *M. edulis* *chilensis*, whereas Ouagajjou et al. [65] in a study with microsatellites considered *M. chilensis* to be a valid species. Analysis of mtDNA and nuclear DNA Me15/16 restriction fragment length polymorphisms (RFLP) revealed the occurrence of native *M. chilensis* and the alleged presence of the native Atlantic blue mussel *M. edulis,* Northern Hemisphere *M. galloprovincialis*, *M. trossulus* genes (not individuals) and hybrids (*M. chilensis* × *M. edulis*) in the region of the Strait of Magellan [66]. Recent studies also consider that *M. chilensis* should be named *M. edulis* *platensis* [59, 67] or *M. platensis* [68]. A genetic analysis of *Mytilus* populations from Argentina and Uruguay using 30 enzyme loci and five DNA markers (*Glu*-*5*, *Fp*-*1*, *Its*, *CoIII* and *Mac*-*1*) showed that native Atlantic South American populations are closely related to North Atlantic *M. edulis* [69]. Because these populations showed characteristic allele frequencies that differed from those of the Northern Hemisphere *M. edulis* at 10 loci, a taxon name *Mytilus edulis* *platensis* was proposed for native mussels from the Atlantic coasts of South America. Consequently, these names have been used in ecology and aquaculture-related publications e.g. [70, 71]. Based on a comparison of single sequences of their mitogenomes, *M. chilensis* and *M. platensis* were placed in the same clade, representing conspecific variants rather than distinct species [72]. In addition, the name *M. planulatus* or *M. e. platensis* was proposed for all native *Mytilus* populations in South America [68].

To clarify the taxonomic status of native populations of smooth shelled mussels from the Pacific coast of South America, Larrain et al. [31] used SNP analyses to characterize *Mytilus* taxa, including a set of reference samples from North America, Europe and New Zealand. According to their results, the Pacific coast South American native mussel is genetically distinct from the reference species *M. edulis*, *M. galloprovincialis* and *M. trossulus*, and should be recognised as *M. chilensis* Hupé 1854 [41]. In our work, we used a similar set of SNPs to characterize, for the first time, populations of smooth shelled mussels *Mytilus* from the coast of South-Western Atlantic and adjacent waters. The aim of our research was to extend the knowledge of the taxonomic status and distribution along the Atlantic coast of South America and Patagonia of native *Mytilus* taxa and to identify areas where they are potentially endangered by hybridization with the invasive *M. galloprovincialis*.

## Methods

### Sample collection and SNP genotyping

**Fig. 1:**
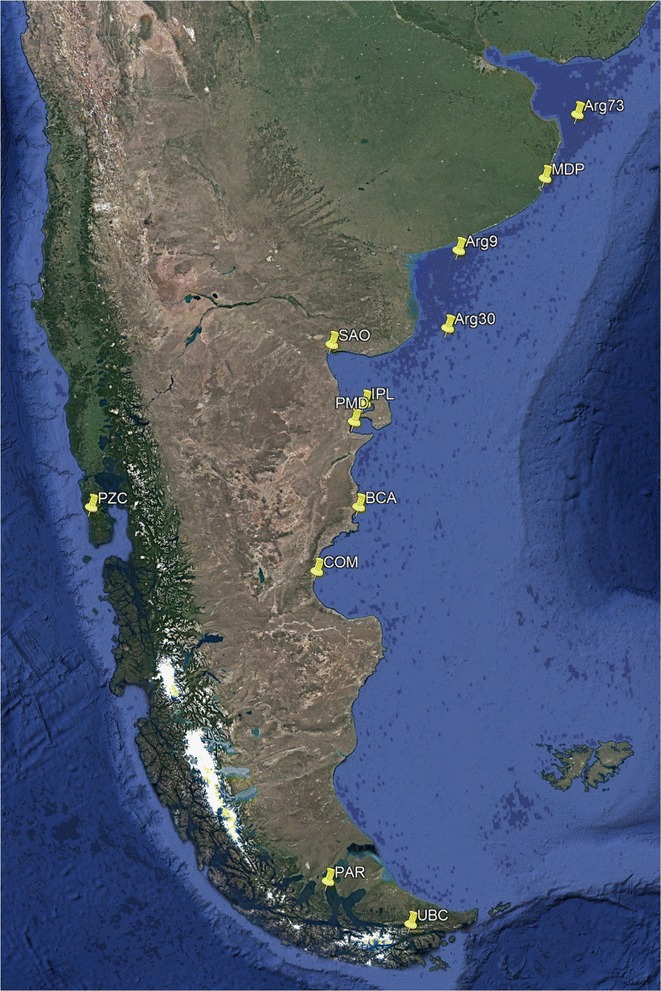
Location of the 12 populations of *Mytilus* from Argentina and Chile, South America (Google Earth Pro). Sampling site names and coordinates are in Table 1

*Mytilus* spp. samples that consisted of 359 individuals of mixed ages and sizes (5 to 50 mm) were collected from ten localities in Argentina and one from Chiloe in Chile between 2012 and 2014 (Fig. 1; Table 1). Specimens or tissue samples were stored in 96% ethanol or at − 70 °C. DNA was isolated from the mantle tissue, using a modified CTAB method according to Hoarau et al. [73]. Eight previously described reference samples were used: *M. edulis* from the Atlantic coast of USA [14] and Northern Ireland [20, 31]; *M. galloprovincialis* from the Northern Hemisphere, i.e. the Atlantic coast of Spain [20, 31] and Mediterranean Sea [20, 29, 31] and from the Southern Hemisphere, i.e. New Zealand [29]; *M. trossulus* from Canada, Halifax (based on hybrid index, [14] and *M. chilensis* from Punta Arenas, Chile [31]. Seventy-nine previously identified SNPs were used [9, 14, 20, 29, 31]. Samples were genotyped using the Sequenom MassARRAY iPLEX genotyping platform [74].

**Table 1:** Detailed information and genetic parameters of the 19 sampled populations of *Mytilus* mussels

Name	Localisation	Country	Number of individuals	$P_{O}$	$H_{O}$	$H_{E}$	Av. gene diversity over loci	Av. number of pairwise differences within population	*F*_IS_	Coordinates		Sampling data
ARG30	Bahia San Blas	Argentina	30	31.37	0.268	0.279	0.079	3.941	− 0.015	41°8′60″S	61°23′59.999″W	2012
ARG73	Buenos Aires	Argentina	30	35.29	0.203	0.232	0.067	3.640	0.085	36°32′24″S	56°11′45.599″W	2012
ARG9	Bahia Blanca	Argentina	30	33.33	0.219	0.245	0.076	3.844	0.091	39°22′30″S	60°33′7.199″W	2012
IPL	Isla de los Pajaros	Argentina	29	33.33	0.297	0.297	0.086	4.532	− 0.035	42°25′16.60″S	64°30′58.99″W	2013
PMD	Puerto Madryn	Argentina	27	60.78	0.379	0.370	0.208	10.334	− 0.066	42°46′58.64″S	64°59′25.13″W	2013
SAO	San Antonio Oeste	Argentina	19	29.41	0.257	0.261	0.070	3.637	− 0.013	40°51′55.50″S	65°3′33.19″W	2012
BCA	Bahia Camarones	Argentina	34	37.25	0.232	0.258	0.093	4.644	0.089	44°45′60.00″S	65°34′0.00″W	2014
COM	Comodoro Rivadavia	Argentina	35	35.29	0.229	0.265	0.089	4.393	*0.117*	45°56′00.00″S	67°32′0.00″W	2014
MDP	Mar del Plata	Argentina	36	41.18	0.196	0.218	0.088	4.413	0.082	37°56′24.00″S	57°35′24.00″W	2014
UBC	Bahia Cauquen, Ushuaia	Argentina	29	33.33	0.311	0.336	0.110	5.593	0.064	54°48′19.09″S	68°17′34.31″W	2013
*Reference samples*												
PZC	Chiloe	Chile	30	39.22	0.275	0.310	0.121	6.055	0.099	42°24′0.54″S	74°10′48.49″W	2012
PAR	Punta Arenas	Chile	33	33.33	0.318	0.335	0.113	5.414	0.021	53° 9′16.12″S	70°54′59.31″W	2012
IRD	Indian River, Delaware	USA	30	47.06	0.239	0.271	0.125	6.263	*0.102*	38°36′27.36′’N	75°3′37.079′’W	2012
LGF	Lough Foyle	Northern Ireland	27	50.98	0.232	0.271	0.103	5.821	0.072	55°5′35.50″N	7°4′48.92″W	2006
CAM	Camarinal	Spain	29	49.02	0.326	0.347	0.158	7.770	0.047	36°4′48.01″N	5°47′58.00″W	2004
ORI	Oristano	Italy	29	50.98	0.285	0.303	0.134	7.049	0.042	39°47′59.88″N	8°31′9.72″E	2006
NZA	Wellington	New Zealand	27	50.98	0.203	0.212	0.097	5.068	0.006	41°22′12.19″S	174°47′39.89″E	2005
AKAR	Akaroa	New Zealand	30	29.41	0.147	0.155	0.042	2.141	0.019	43°40.323′S	172°57.914′E	2008
KKAT	Halifax	Canada	28	56.86	0.204	0.246	0.121	6.396	*0.131*	44°30′33.79″N	63°29′24.91″W	2003

$P_{O}$, % of polymorphic loci; *F*_IS_, inbreeding coefficient; $H_{O}$, observed heterozygosity; $H_{E}$, expected heterozygosity; values with P < 0.05 after Benjamini–Yekutieli correction are marked in italic

### Data analysis

Arlequin v.3.5.1.2 [75] was used to estimate allele frequencies, the proportion of polymorphic SNPs ($P_{O}$), minor allele frequency (MAF), genetic diversity, observed ($H_{O}$) and expected ($H_{E}$) heterozygosity values for each locus and population. In addition, the statistical significance of the inbreeding coefficient *F*_IS_ (> 0) was tested by 10,000 permutations of alleles between individuals. Departures from Hardy–Weinberg equilibrium (HWE) were tested by exact test, and significance was determined by Markov chain Monte Carlo simulations. The most informative loci were detected by identifying *F*_ST_ outlier loci. The upper limits of the 95% confidence interval were identified with 20,000 iterations and characterized by estimating the 0.05 and the 0.95 and 0.99 quantiles of the distribution. The false discovery rate (FDR-BY) was applied to correct significance (P) values after multiple testing [76, 77]. Genetic differentiation between populations was determined based on allele frequencies of SNPs using pairwise *F*_ST_ values in Arlequin. The *F*_ST_ distance measures in the Newick format, which were obtained from SNP allele frequencies data in POPTREEW [78] were used to construct a neighbour-joining (NJ) tree with the MEGA software version 6 [79]. Robustness of relationships was assessed using 10,000 bootstrap replicates. We used two methods for the population structure analyses. First, clustering analysis was carried out with the STRUCTURE v. 2.3.4 software [80, 81]. STRUCTURE was used under a model that assumes admixture, ignores population affiliation and allows for the correlation of allele frequencies between clusters. The admixture model used in this analysis allows individuals to have mixed ancestry, i.e. fractions of the genome can originate from different ancestors. The number of genetic clusters (*K*) was estimated by computing likelihood over 10 runs for values of *K* ranging from 1 to the number of populations studied plus 1. At the plateau of the graph curve, the value of *K* captures the main structure of the populations. The best-fit number of genetic clusters was determined by calculating the logarithmic probability LnP(K) using the ΔK method [82]. Threshold q-values of 0.2 were used as a criterion to separate hybrids and pure mussels [83]. Individuals were considered residents if their q values were higher than 0.8 in the area where they were sampled. Individuals with q-values from 0.2 to 0.8 were considered to be potentially admixed, since they could not be readily assigned as residents or migrants [84]. A Markov chain Monte Carlo simulation was run for 100,000 iterations following a burn-in period of 50,000 iterations.

Correspondence analysis (CA) [85] implemented in GENETIX [86], was used to visualize the genetic substructure at the population and individual levels. The results are presented as a scatter plot, with the axes representing the contribution of inertia of the data matrix in a way that is analogous to the total variance in allelic frequency. Genetic assignment was obtained by using two methods. Following the STRUCTURE analysis, each individual was assigned with high confidence when q was higher or equal to 0.8 for a single cluster. In the second method, assignment of individuals to population of origin was obtained by using the frequency criteria on the basis of multilocus genotype data [87] in a self-assignment test with the leave-one-out (LOO) procedure implemented in GeneClass2.0 [88]. Individuals were considered to be correctly assigned to their location of origin if the assignment probability to that group was higher than any other assignment probability to any other group.

Following preliminary analyses that indicated that the Argentinian population of Puerto Madryn (PMD) contained possible hybrids, the software NewHybrids v1 [89] was used to estimate the posterior probability that individuals from PMD fell into each of the six genotypic categories (or classes corresponding to hybrid categories): native *M. platensis*, *M. galloprovincialis*, F1 hybrids, F2 hybrids and two types of backcrosses.

## Results

### SNP validation, genetic diversity and Hardy–Weinberg equilibrium

Of the 79 SNPs assayed, 55 were successfully genotyped with an acceptable quality score, and among these, 51 were polymorphic for 562 mussels from 19 samples [see Additional file 1: Table S1]. Of these 51 SNPs, 46 (90.2%) were located in coding regions, among which only three were non-synonymous and five (9.8%) were located in non-coding regions. Six loci were polymorphic in mussels from all samples [see Additional file 2: Table S2]. MAF per locus ranged from 0 to 0.362 (BM32A) with a mean (± SD) of 0.078 ± 0.091 across all loci. Only five loci in single populations were not in Hardy–Weinberg equilibrium (HWE) after correction for multiple testing.

*F*_ST_ values at individual SNPs ranged from 0.014 to 1 [see Additional file 1: Table S1]. Twenty-three SNPs had *F*_ST_ values significantly different from zero. Several groups of samples were tested to detect highly informative SNPs that are effective for differentiation between groups. Populations and individuals (hybrids) with an admixture of different groups (taxa) were excluded from the analysis. An outlier test was carried out for the group of eight populations from Argentina (without PMD and UBC) and the group of reference samples from Chile, USA, Canada, Northern Ireland, Spain, Italy and New Zealand. The test indicated 14 outlier loci, of which seven were characteristic only for *M. trossulus* and seven (BM101A, BM106B, BM12A, BM151A, BM17B, BM21B and BM6C) that differentiated the Argentinian samples. These latter same seven SNPs were identified as outliers in a test between *M. platensis* and *M. galloprovincialis* samples.

Four SNPs were effective at differentiating between the Argentinian and Chilean populations, and among these, two were significant also in the outlier analysis (BM151A and BM21B) while BM203C and BM57A were new. Subsequently, an outlier test was carried out to detect SNPs that could differentiate populations from Argentina and *M. edulis* samples from America and Europe. This test highlighted six outlier SNPs (BM106B, BM12A, BM12C, BM21B, BM21C and BM5D), of which three had not been previously reported. These findings show that 19 SNPs are sufficient to differentiate Argentinian populations from those of all other regions and highlighted their distinct taxon status. Five SNPs were identified as highly informative (P < 0.01): BM106B, BM12A, BM151A, BM21B, and BM6C.

### Genetic diversity

The proportion of polymorphic SNPs ($P_{o}$) ranged from 29.4 to 60.8% between populations, the lowest proportions being observed in most of the Argentinian populations. The values of $P_{o}$, $H_{o}$, gene diversity within populations were highest for the Argentinian PMD population, in which individuals of mixed origin were observed (Table 1). Based on the *F*_IS_ measures (averaged across all polymorphic loci in each population), an excess of homozygotes was found only in one population from Argentina (COM) and two reference samples (IRD, KKAT).

### Genetic variation and differentiation between populations

**Fig. 2:**
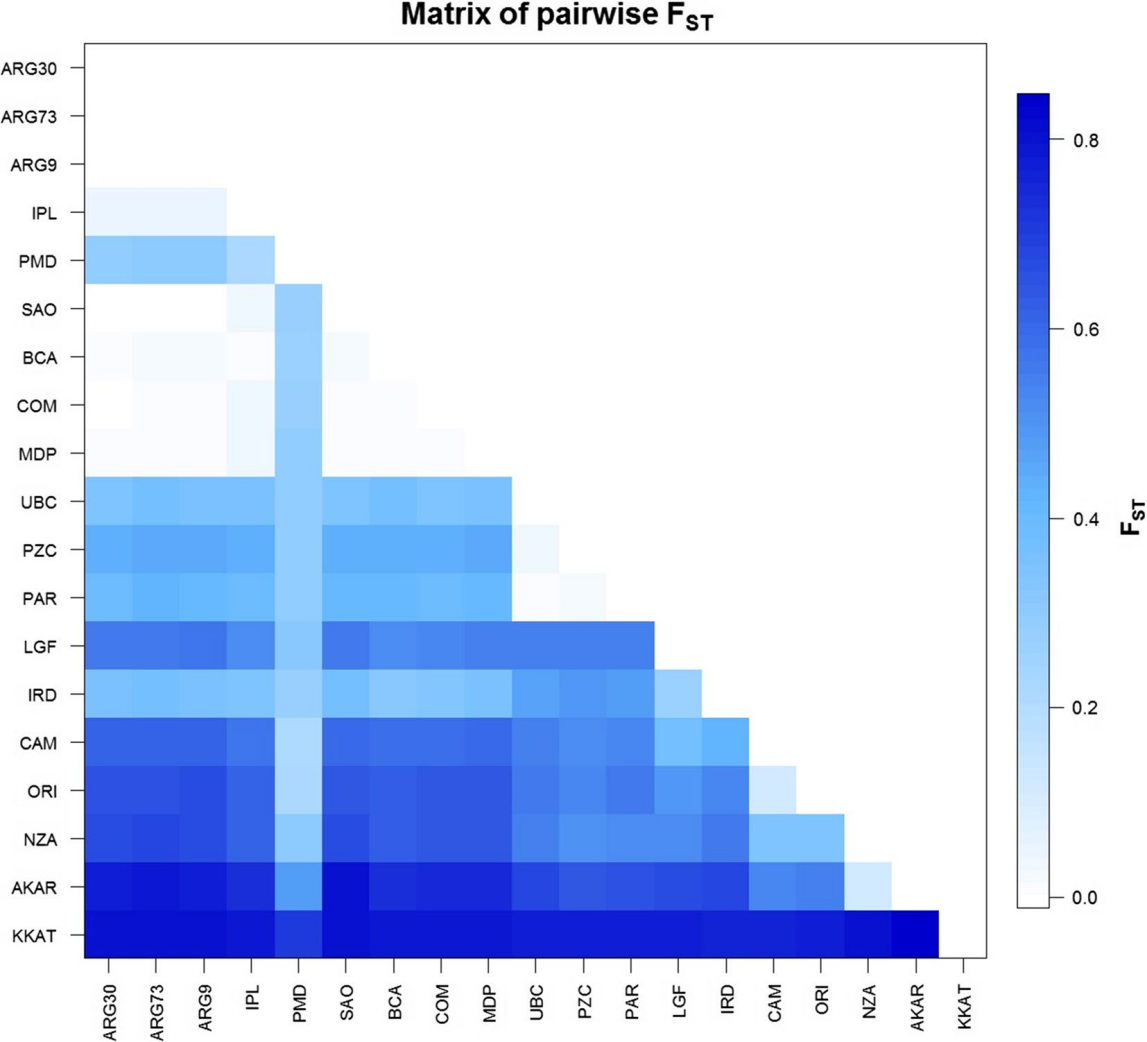
Matrix of pairwise *F*_ST_ for 19 *Mytilus* spp. samples based on 51 SNPs

We constructed a neighbour-joining tree based on *F*_ST_ distance measures to detect the genetic relationships within 19 samples [see Additional file 3: Figure S1]. This identified five groups of populations i.e. *M. trossulus*, *M. edulis*, Northern and Southern Hemisphere *M. galloprovincialis*, *M. chilensis* and *M. platensis*, and one sample (PMD) that exhibited admixture in the *Mytilus* taxa. The population from Ushuaia (UBC) clustered with *M. chilensis*. Internal branches were short between *M. chilensis* and *M. platensis* populations, whereas they were long between *M. edulis* and *M. galloprovincialis* populations. Pairwise *F*_ST_ values were significantly different from zero after FDR-BY correction between most pairs of samples (Fig. 2) and [see Additional file 4: Table S3]. The largest differences were observed between *M. trossulus* and southern *M. galloprovincialis* from Akaroa, New Zealand (reaching values as high as 0.84). Pairwise *F*_ST_ estimates between the seven populations from Argentina (*M. platensis*) were not significantly different from zero (0 to 0.025), which indicated that these seven populations are mostly homogenous.

### Population structure

**Fig. 3:**
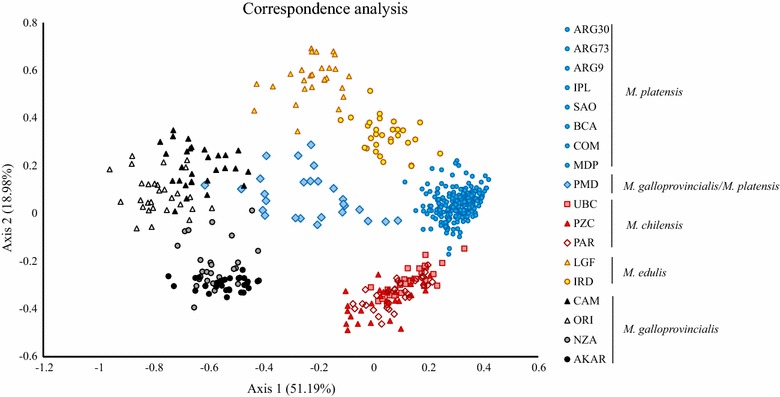
The first two axes of the correspondence analysis (CA) computed from the SNP data on ten populations from Argentina and reference populations of *M. edulis*, *M. galloprovincialis* and *M. chilensis* from America, Europe and New Zealand. Each dot (point) is an individual

To characterize the population structure of *Mytilus*, correspondence analyses (CA) were carried out on 18 samples of *M. edulis*, *M. galloprovincialis*, *M. chilensis* and *M. platensis*, by excluding *M. trossulus* for higher resolution (Fig. 3). Figure 3 shows a clear separation between *M. galloprovincialis* and the other samples along axis 1. *M. platensis* and *M. chilensis* individuals formed very tight groups, in contrast to the *M. edulis*, *M. galloprovincialis* and most PMD individuals, which displayed more dispersion. The PMD sample occupied a central position between all other samples and overlapped only with *M. galloprovincialis* from the Atlantic coast of Europe (CAM).

**Fig. 4:**
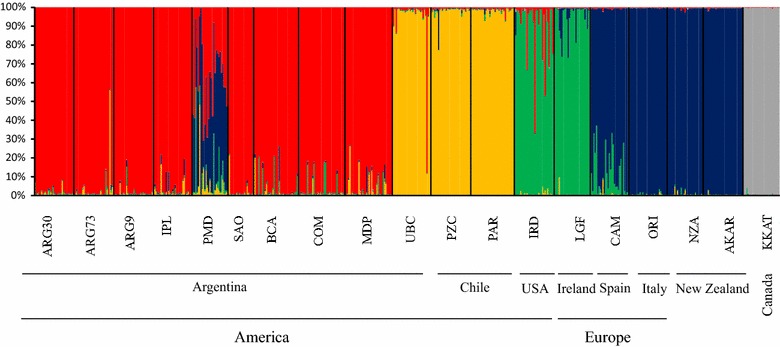
Structure plots for the 19 studied populations (K = 5). Each individual is represented by a single vertical line broken into five coloured segments, with lengths proportional to each of the K inferred clusters. Abbreviation of the samples is provided in Table 1. Vertical black lines separate the populations

STRUCTURE analysis showed that the LnP(D) increase was largest (i.e. ΔK was highest) for *K* = 3, and then *K* = 5. This result indicated that the best-fit number of genetic clusters was found for *K* = 3, for which differentiation between clusters corresponding to *M. trossulus*, *M. galloprovincialis* and all other *Mytilus* taxa occurred. The high value of ΔK for *K* = 5 suggested further subdivision, with five clusters corresponding to five taxa: *M. trossulus*, *M. galloprovincialis*, *M. edulis*, *M. chilensis* and *M. platensis* (Fig. 4). These results confirmed the close relationship between *M. edulis*, *M. chilensis* and *M. platensis* taxa.

### Assignment of individuals

Using the panel of 51 SNPs, two methods of analysis were used to test the assignment of individuals from Argentina to the most likely population and regions, based on reference taxa. In the STRUCTURE analysis at *K* = 5, most of the 562 individuals were properly assigned to their original samples with a genome admixture value q higher than 0.8 [see Additional file 5: Table S4]. Eight populations from Argentina clustered together (*M. platensis*), whereas the sample from Ushuaia (UBC) clustered with the two *M. chilensis* samples from Chile. A few exceptions were observed: one individual from UBC was assigned to *M. platensis*, single individuals from ARG73 and IRD were considered potentially admixed (*M. chilensis* × *M. platensis* and *M. platensis* × *M. edulis*, respectively). Furthermore, the entire PMD population from Argentina showed very high levels of admixture: one individual was assigned to *M. galloprovincialis* with q higher than 0.8, whereas all other individuals were ambiguously assigned to two clusters, mainly to *M. galloprovincialis* and *M. platensis*, with q ranging from 0.2 to 0.8. In addition, two individuals were assigned to two other clusters *M. edulis* and *M. galloprovincialis*, and *M. chilensis* and *M. galloprovincialis*. In general, most individuals had the highest q for the *M. galloprovincialis* cluster followed by the *M. platensis* cluster.

Individuals were assigned to baseline populations based on region of origin with a success rate of 97.8% using GeneClass2 [see Additional file 5: Table S4]. We performed a new (second) assignment analysis of the PMD population after removing the PMD sample from the baseline populations to identify the region of origin and found that 45% of the individuals from Puerto Madryn were assigned to *M. galloprovincialis* from the Mediterranean and Atlantic populations and more than 30% to the Argentinian population from Isla de los Pajáros (IPL). Four individuals were placed in the *M. edulis* population from North America and, for one individual the highest probability occurred for *M. chilensis* populations from Chile (PZC).

### Identification of hybrids

Because most of the individuals from PMD were assigned mainly to two clusters using STRUCTURE analysis, *M. platensis* and Northern Hemisphere *M. galloprovincialis*, we carried out additional analyses to identify the type of hybrid (F1 or F2). Using the program NewHybrids, 19 SNPs that are effective at differentiating between populations from Argentina were used to analyze all the Argentinian populations and three groups of reference samples: *M. platensis*, Northern Hemisphere *M. galloprovincialis*, and *M. chilensis*. All PMD individuals were identified as hybrids except one that was classified as *M. galloprovincialis* and F2 hybrids that carry *M. platensis* and *M. galloprovincialis* alleles were detected in 88.89% of the PMD individuals with a probability higher than 90%. We did not identify any F1 hybrids and detected only one backcross (probability of ~ 60%) to *M. platensis*. In addition, using *M. chilensis* as a reference sample, two F2 hybrids that carry *M. chilensis* and *M. galloprovincialis* (PMD) alleles and *M. chilensis* and *M. platensis* (ARG73) alleles were identified.

### Identification of *Mytilus* taxa

**Fig. 5:**
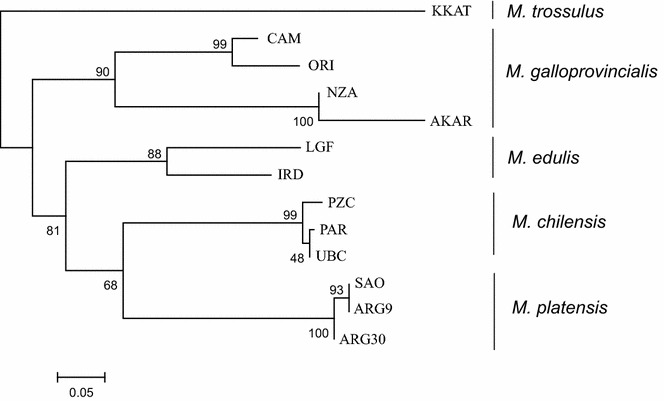
Neighbour-joining tree of *Mytilus* spp. samples from Argentina and reference populations of *M. edulis*, *M. trossulus*, *M. galloprovincialis* and *M. chilensis* from America, Europe and New Zealand based on the *F*_ST_ distance measures obtained with POPTREEW and visualised with MEGA version 6. NJ tree constructed using individuals without admixture (q > 0.8 or q < 0.2) identified by STRUCTURE analysis (K = 5)

To determine the degree of similarity between taxa, we compared the different groups of populations without hybrids (admixture individuals). We constructed a NJ tree by using only non-admixed individuals as identified by STRUCTURE and GeneClass2 analysis, which revealed five well-supported clades that coincide with five separate taxa: *M. trossulus*, *M. galloprovincialis*, *M. edulis*, *M. chilensis* and *M. platensis* (Fig. 5). Based on pairwise *F*_ST_ values, *M. platensis* samples differed from *M. chilensis* samples (*F*_ST_ = 0.421) and *M. edulis* samples (*F*_ST_ ranging from 0.395 for American to 0.552 for European individuals) [see Additional file 6: Table S5]. *M. platensis* samples differed from Mediterranean *M. galloprovincialis* (*F*_ST_ = 0.65). The level of differentiation observed between *M. platensis* and *M. edulis* was comparable to that between *M. edulis* and Atlantic *M. galloprovincialis* (*F*_ST_ = 0.427, on average) and was highest between *M. platensis* and *M. trossulus* and Southern Hemisphere *M. galloprovincialis* taxa (*F*_ST_ values as high as 0.797).

Overall, these results indicate that the samples of *Mytilus* in Argentina were composed of three groups: (1) eight samples of native *M. platensis*, (2) a southern group represented by the UBC population of native Chilean blue mussel (*M. chilensis*), and (3) the population from Puerto Madryn (PMD) composed of hybrid individuals (mostly between *M. galloprovincialis* and *M. platensis*). Argentinian populations showed a low level of hybridization, with the exception of the PMD population, which is composed mainly of hybrid individuals.

## Discussion

In most previous studies of South American smoothed-shelled blue mussel *Mytilus* populations e.g. [59, 68], only a small number of nuclear DNA markers, allozymes or mtDNA markers with limited resolution power was used. Recently, the efficiency of SNP analyses to study the genetic characteristics of hatchery and wild populations has been reported [90]. In our study, we carried out a thorough population genetic analysis and, for the first time, we show that *Mytilus* populations from the Atlantic coast of South America and Isla Grande de Tierra del Fuego are strongly differentiated and belong to at least three taxa. Native *M. platensis* is a separate taxon from other *Mytilus* species, including native *M. chilensis*. *M. platensis* predominates on the Argentinian coast south of Rio de La Plata, whereas *M. chilensis* inhabits the southernmost part of the South American coasts including the area of Isla Grande de Tierra del Fuego. Invasive European *M. galloprovincialis* and introgressed specimens were found in one location (PMD). These findings shed new light on the genetic relationships between South American *Mytilus* taxa. *M. platensis* was first described by d’Orbigny in 1846 [40] based on contemporary and paleontological samples that were collected from the area of Maldonaldo, Uruguay (Rio de la Plata) on the Atlantic coast. Based on ecological studies of coastal ecosystems and on toxicology analyses in fisheries and aquaculture of smooth shelled mussels, the presence of *M. platensis* (*M. e. platensis*) was reported on the Atlantic coast, from Uruguay including Maldonaldo in the north [45, 91–93] and at Mar del Plata on artificial reefs [94] to Golfo San Matias, Bahía San Julián, Santa Cruz Province and the Rio Gallegos Estuary, Patagonia, in the south [51, 54, 95, 96]. Furthermore, occurrence of *M. chilensis* (*M. e. chilensis*) on the Argentinian coast of the Isla Grande de Tierra del Fuego including the Ushuaia Bay was tentatively assumed from studies on similar topics [97–99].

In our study, we detected only one native individual *M. chilensis* × *M. platensis* hybrid and one *M. platensis* × *M. edulis* hybrid in more northern sampling sites. Oyarzún et al. [66] reported the occurrence of large numbers of hybrids between *M. chilensis* and native Atlantic *Mytilus* in the Magellan Region (southern Chile). However, since they used molecular markers with lower diagnostic power for Southern Hemisphere *Mytilus* taxa, their results cannot be directly compared with those reported here. We observed no hybridization in populations of *M. chilensis* from neighbouring sampling sites (Punta Arenas and Ushuaia) and detected only pure individuals of *M. chilensis* and one *M. platensis* individual in the area of the Isla Grande de Tierra del Fuego.

Based on the use of single molecular markers, hybridization of invasive Northern Hemisphere *M. galloprovincialis* with Southern Hemisphere native mussels in Chile, New Zealand and Australia was reported [28, 99, 100] and recently confirmed using SNP genotyping for populations from New Zealand and Chile [29, 31]. We used almost the same set of SNPs to investigate ten populations from Argentina. Our findings point to a local invasion and colonization event. No deficiency of heterozygotes was found within the PMD population, which suggests the absence of reproductive barriers. This contrasts with analyses on hybrid populations from Scotland, Barents Sea, Norway and Greenland for which there is a deficiency of heterozygotes [14, 20].

The introduction of *M. galloprovincialis* from the Northern Hemisphere might be related to past aquaculture activities in the Nuevo Gulf, although Dellatorre et al. [51] clearly stated that the cultivated mussels in a long-line system in Argentina originate from a natural settlement of *M. e. platensis*. In addition, the National authorities in Argentina did not issue any permission for any experiment related to the introduction of *M. galloprovincialis* (Marcela Alvarez, Subsecretaría de Pesca de la Nación, personal communication). It was further confirmed that no related aquaculture experiments were performed in Argentina (Mario Lasta, Instituto Nacional de Investigación y Desarrollo Pesquero, Mar Del Plata, personal communication). Another more likely scenario is the accidental introduction of *M. galloprovincialis* by means of ship transportation. Fishing vessels as well as big cruise ships enter Puerto Madryn from Europe, North America and Chile [101] with the problems of biofouling (accumulation of aquatic microorganisms, plants and animals on hull submerged surfaces) and the discharge of ballast waters [102], which releases these organisms in the environment. A previous study reported a high percentage of exotic and invasive species including unidentified *Mytilus* sp. in the Puerto Madryn local ecosystem [103]. For example, the Mytilid *Semimytilus algosus* was transported to Puerto Madryn (Nuevo Gulf) by a fishing vessel and released during in-water hull cleaning [104], which constitutes an example of introduction of a species similar to *M. galloprovincialis*.

The presence of Southern Hemisphere *M. galloprovincialis* was detected in Punta Arenas and named M. *galloprovincialis planulatus* Lmk 1819 [57]. However, Lamarck [105] originally described *M. planulatus* from Port du Roi George, Nouvelle-Hollande (present day Albany, Australia) in 1819. To confirm or not its occurrence in South America, new genetic markers such as SNPs could be used. However, our results did not confirm its presence in the studied area and a study of *Mytilus* populations from Chile, including Punta Arenas did not detect it [31]. Therefore, there is no evidence supporting the potential existence of *M. planulatus* in South America [68].

## Conclusions

We have demonstrated that *M. platensis* (or *M. e. platensis*) and *M. chilensis* are differentiated and native taxa in South America, which indicates a distinct evolutionary history of *Mytilus* taxa from the Atlantic and Pacific coasts. Our analysis identified 19 SNPs (five of which are highly informative) that are effective in differentiating populations from Argentina. *M. platensis* is endangered by hybridization with the introduced European *M. galloprovincialis* in one area in Argentina (Puerto Madryn), presumably due to accidental introduction from ship traffic. We also confirmed the occurrence of *M. chilensis* on the Isla Grande de Tierra del Fuego, southern coast of Argentina. Knowledge of the origin of the mussels is very important for the conservation of native populations in the context of aquaculture activities in this area. The occurrence of the *M. platensis* × *M. galloprovincialis* hybrid population in Puerto Madryn that demonstrates one of the most invasive species, Northern Hemisphere *M. galloprovincialis*, is a threat to native populations. Continued monitoring is needed to check for the spread between these two taxa on the Atlantic coast of South America.

## Additional files

**Additional file 1: Table S1.** SNP polymorphisms in populations of *Mytilus* spp. studied. Description: Information is presented on SNP properties, genome location, substitution type, *F*_ST_ *P* value associated with test for outlier status, minor allele frequency, GenBank annotation and references.

**Additional file 2: Table S2.** Allele frequencies of 51 SNPs for 19 *Mytilus* spp. sample. Description: The data show frequencies of all alleles at the studied SNP loci in all samples.

**Additional file 3: Figure S1.** Neighbour-joining tree of native South American and the reference *Mytilus* taxa. Description: Neighbour-joining tree shows genetic relationship between 19 *Mytilus* spp. samples from Argentina and reference populations of *M. edulis*, *M. trossulus*, *M. galloprovincialis* and *M. chilensis* from America, Europe and New Zealand based on the *F*_ST_ distance measures obtained with POPTREEW and visualised with MEGA version 6.

**Additional file 4: Table S3.** *F*_ST_ distance matrix for 19 *Mytilus* spp. samples for 51 SNPs. Description: Values of *F*_ST_ with P < 0.05 after Benjamini–Yekutieli (FDR-BY) correction are marked in bold. Site names and locations are in Table 1.

**Additional file 5: Table S4.** Result of population assignment algorithms STRUCTURE and GeneClass for 19 populations of mussels. Description: Two methods of analysis were used to test the assignment of individuals from Argentina to the most likely population and regions, based on reference taxa. In the STRUCTURE analysis at *K* = 5, most individuals were properly assigned to their original samples. Individuals were assigned with GeneClass2 to baseline populations based on region of origin with a success rate of 97.8%.

**Additional file 6: Table S5.** *F*_ST_ distance matrix for *Mytilus* spp. samples. Description: *F*_ST_ distance matrix is presented for *Mytilus* spp. samples from Argentina and reference populations of *M. edulis*, *M. trossulus*, *M. galloprovincialis* and *M. chilensis*, obtained with POPTREEW for individuals without admixture (q > 0.8 or q < 0.2) identified by STRUCTURE analysis.
