# Supplementary material for: Single nucleotide polymorphisms in native South American Atlantic coast populations of smooth shelled mussels: hybridization with invasive European Mytilus galloprovincialis
Source: Genet Sel Evol. 2018 Feb 22;50:5. doi: 10.1186/s12711-018-0376-z (PMC5824471; doi:10.1186/s12711-018-0376-z)
Supplement: Supplementary file 1 — Additional file 1: Table S1. SNP polymorphisms in populations of Mytilus spp. studied. Description: Information is presented on SNP properties, genome location, substitution type, FST P value associated with test for outlier status, minor allele frequency, GenBank annotation and references. [file 12711_2018_376_MOESM1_ESM.pdf]

Table S1. SNP properties, genome location, substitution type, FST P-value associated with test for outlier status, minor allele frequency, GenBank annotation and references.

	Locus name	Location	Region	Substitution	Allele	Frequent allele	FST	B-Y	H_O	F_{IS}	Nº of locations where the locus was polymorphic	MAF	GenBank annotation	Reference
1	BM101A	Ribosomal protein L7a	coding	synon	A/T	T	0.748		0.435	-0.006	8	0.084	KT713378	Wenne et al. 2016
2	BM102A	Proteasome subunit beta type-6-like	coding	synon	C/T	T	**0.145**	*	0.248	-0.026	16	0.143	KT713379	Wenne et al. 2016
3	BM103B	Proteasome subunit beta type-5-like	coding	nonsyn	A/G	A	0.737		0.074	0.053	1	0.014	KT713380	Wenne et al. 2016
4	BM105A	UnKnown	NA	NA	A/G	A	0.377		0.136	0.068	6	0.067	KT713381	Wenne et al. 2016
5	BM106B	UnKnown	NA	NA	A/G	G	0.787		0.417	**0.980**	8	0.056	KT713382	Wenne et al. 2016
6	BM10B	Ribosomal protein S20	coding	synon	A/C	A	0.805		0.081	0.027	1	0.010	KJ871040	Zbawicka et al.. 2014
7	BM113A	Protein BTG1	coding	synon	A/T	A	**1**	*	1.000	0	0	0.000	KT713385	Wenne et al. 2016
8	BM118A	UnKnown	NA	NA	A/G	G	0.139		0.055	-0.027	5	0.029	KT713388	Wenne et al. 2016
9	BM11A	Ribosomal protein L22	coding	synon	A/G	A	**0.982**		0.098	0.000	1	0.001	KJ871041	Zbawicka et al.. 2014
10	BM12A	Ribosomal protein L23a	coding	synon	C/T	T	**0.875**		0.454	0.092	7	0.038	KJ871042	Zbawicka et al.. 2014
11	BM12C	Ribosomal protein L23a	coding	synon	C/T	C	0.628		0.417	0.096	14	0.100	KJ871042	Wenne et al. 2016
12	BM147A	UnKnown	NA	NA	C/T	C	**0.434**	*	0.380	-0.004	17	0.141	KT713383	Wenne et al. 2016
13	BM151A	adhesive foot protein gene	coding	synon	G/T	G	**0.865**		0.434	**0.178**	9	0.033	HQ257471.1	Gardner et al. 2016
14	BM16B	ribosomal protein L21	coding	synon	C/T	A	**0.014**	*	0.280	-0.035	19	0.167	KJ871044	Wenne et al. 2016
15	BM17B	Ribosomal protein L7a	coding	synon	A/G	A	0.684		0.477	0.100	11	0.113	KJ871045	Zbawicka et al.. 2014
16	BM201B	*H4 histone* gene	noncoding (intron)	NA	A/C	C	0.704		0.070	**0.727**	1	0.015	AY267750.1	Zbawicka et al.. 2012
17	BM201C	*H4 histone* gene	coding	synon	G/T	T	0.513		0.243	-0.039	8	0.093	AY267750.1	Zbawicka et al.. 2012
18	BM202A	*H3 histone* gene	noncoding (intron)	NA	A/C	A	**1**	*	1.000	0	0	0.000	AY267749.1	Zbawicka et al.. 2012
19	BM202B	*H3 histone* gene	coding	synon	A/T	T	**1**	*	1.000	0	0	0.000	AY267749.1	Zbawicka et al.. 2012
20	BM203B	*H2B histone* gene	noncoding (intron)	NA	C/T	C	0.492		0.241	-0.030	8	0.095	AY267742.1	Zbawicka et al.. 2012
21	BM203C	*H2B histone* gene	noncoding (intron)	NA	C/T	A	0.622		0.530	0.064	18	0.131	AY267742.1	Zbawicka et al.. 2012
22	BM203D	*H2A histone* gene	noncoding (intron)	NA	A/T	A	**1**	*	1.000	0	0	0.000	AY267757.1	Zbawicka et al.. 2012
23	BM204A	*p53*	coding	synon	C/T	T	**0.333**		0.169	0.023	7	0.080	DQ865151	Zbawicka et al.. 2012
24	BM21B	qm-like protein	coding	nonsyn	C/G	C	0.726		0.491	-0.071	13	0.104	KJ871047	Zbawicka et al.. 2014
25	BM21C	qm-like protein	coding	synon	A/C/T	A	0.652		0.137	-0.092	8	NA	KJ871047	Zbawicka et al.. 2014
26	BM26B	UnKnown13	NA	NA	A/T	A	0.794		0.126	-0.102	4	0.015	KJ871050	Zbawicka et al.. 2014
27	BM2G	UnKnown05	coding	synon	G/T	G	0.944		0.095	-0.042	1	0.003	KJ871032	Zbawicka et al.. 2014
28	BM30A	Ribosomal protein l17	coding	synon	A/G	A	0.856		0.086	-0.150	1	0.008	KJ871052	Zbawicka et al.. 2014
29	BM30C	Ribosomal protein l17	coding	synon	A/T	T	0.51		0.052	0.087	1	0.025	KJ871052	Zbawicka et al.. 2014
30	BM32A	ubiquinol-cytochrome c reductase subunit 6	coding	synon	A/G	G	**0.098**	*	0.496	**0.086**	19	0.362	KT713371	Wenne et al. 2016
31	BM33B	Cytochrome c oxidase subunit IV	coding	synon	A/T	A	0.398		0.051	-0.130	3	0.027	KJ871054	Zbawicka et al.. 2014
32	BM35C	Ribosomal protein L7	coding	synon	A/T	A	**0.37**	*	0.488	0.049	19	0.223	KJ871055	Wenne et al. 2016
33	BM36F	ribosomal protein S3a	coding	synon	A/C	A	0.777		0.129	0.081	6	0.016	KT713373	Wenne et al. 2016
34	BM38B	ribosomal protein S8e	coding	synon	A/G	G	**0.255**	*	0.381	0.010	19	0.180	KT713368	Wenne et al. 2016
35	BM44B	ubiquitin/ribosomal protein S27a	coding	synon	A/G	G	0.526		0.053	**0.492**	1	0.024	KJ871057	Zbawicka et al.. 2014
36	BM50B	CoA-binding protein	coding	synon	A/G	G	0.056		0.007	-0.060	1	0.004	KJ871059	Zbawicka et al.. 2014
37	BM54A	ETC_C1_NDUFA4	coding	synon	A/G	A	**1**	*	1.000	0	0	0.000	KJ871060	Zbawicka et al.. 2014
38	BM57A	NADH-ubiquinone_oxidoreductase	coding	nonsyn	C/T	C	0.523		0.341	**0.130**	11	0.120	KT713374	Wenne et al. 2016
39	BM57D	NADH-ubiquinone_oxidoreductase	coding	synon	A/C	C	0.445		0.152	0.000	4	0.072	KT713374	Wenne et al. 2016
40	BM5B	Ribosomal protein S6e	coding	synon	A/G	A	0.069		0.027	-0.083	3	0.014	KJ871035	Wenne et al. 2016
41	BM5D	Ribosomal protein S6e	coding	synon	C/T	C	**0.263**		0.141	-0.067	8	0.071	KJ871035	Zbawicka et al.. 2014
42	BM61A	Ribosomal_L1	coding	synon	C/T	T	**0.316**	*	0.502	**0.270**	18	0.247	KT713375	Wenne et al. 2016
43	BM62A	Ribosomal L13e	coding	synon	A/G	G	0.664		0.152	0.168	6	0.028	KJ871064	Zbawicka et al.. 2014
44	BM64A	Ribosomal protein L35	coding	synon	C/T	C	**1**	*	1.000	0	0	0.000	KJ871065	Zbawicka et al.. 2014
45	BM67C	Ribosomal protein S6e	coding	synon	A/T	T	**0.292**	*	0.477	**0.118**	18	0.273	KJ871066	Wenne et al. 2016
46	BM6C	EFG_N	coding	synon	C/T	C	0.626		0.512	0.059	13	0.133	KJ871036	Zbawicka et al.. 2014
47	BM78B	UnKnown12	coding	synon	A/G	G	0.592		0.380	0.057	15	0.104	KJ871069	Zbawicka et al.. 2014
48	BM8E	Ribosomal protein L3	coding	synon	A/G	G	**0.966**		0.103	-0.001	2	0.002	KJ871038	Zbawicka et al.. 2014
49	BM92B	UnKnown06	coding	synon	A/T	A	**1**	*	1.000	0	0	0.000	KJ871074	Zbawicka et al.. 2014
50	BM9B	Ribosomal protein S2	coding	synon	A/G	G	**0.143**	*	0.496	**0.099**	19	0.352	KJ871039	Zbawicka et al.. 2014
51	BM9C	Ribosomal protein S2	coding	synon	A/C/T	A	**0.212**	*	0.544	0.059	19	NA	KJ871039	Zbawicka et al.. 2014

Values with P < 0.05 are marked in bold; *, values with P < 0.05 after Benjamini–Yekutieli correction; $F_{IS,}$ inbreding coefficient; $H_{O,}$ observed heterozygosity; NA, not applicable
