# Supplementary material for: Single nucleotide polymorphisms in native South American Atlantic coast populations of smooth shelled mussels: hybridization with invasive European Mytilus galloprovincialis
Source: Genet Sel Evol. 2018 Feb 22;50:5. doi: 10.1186/s12711-018-0376-z (PMC5824471; doi:10.1186/s12711-018-0376-z)
Supplement: Supplementary file 2 — Additional file 2: Table S2. Allele frequencies of 51 SNPs for 19 Mytilus spp. sample. Description: The data show frequencies of all alleles at the studied SNP loci in all samples. [file 12711_2018_376_MOESM2_ESM.pdf]

TableS2. Allele frequencies of 51 SNP loci for 19 Mytilus spp. populations

Locus	Allele		ARG30	ARG73	ARG9	IPL	PMD	SAO	BCA	COM	MDP	UBC	PZC	PAR	IRD	LGF	CAM	ORI	NZA	AKAR	KKAT
BM101A	1	A	0	0	0	0.0714	0.5385	0	0.0147	0	0.0139	0.2414	0.4667	0.2879	0	0.0417	1	1	1	1	0
BM101A	2	T	1	1	1	0.9286	0.4615	1	0.9853	1	0.9861	0.7586	0.5333	0.7121	1	0.9583	0	0	0	0	1
BM102A	1	C	0.1167	0.0862	0.1207	0.0345	0.0769	0.0526	0.0735	0.1	0.0139	0.2414	0.3833	0.1818	0.4667	0.3125	0.3393	0.12	0	0	0
BM102A	2	T	0.8833	0.9138	0.8793	0.9655	0.9231	0.9474	0.9265	0.9	0.9861	0.7586	0.6167	0.8182	0.5333	0.6875	0.6607	0.88	1	1	1
BM103B	1	A	1	1	1	1	1	1	1	1	1	1	1	1	1	1	1	1	1	1	0.2593
BM103B	2	G	0	0	0	0	0	0	0	0	0	0	0	0	0	0	0	0	0	0	0.7407
BM105A	1	A	1	1	1	1	0.7407	1	1	1	0.9861	1	1	1	1	0.9815	0.431	0.5714	0.8704	1	1
BM105A	2	G	0	0	0	0	0.2593	0	0	0	0.0139	0	0	0	0	0.0185	0.569	0.4286	0.1296	0	0
BM106B	1	A	0	0.0333	0.0333	0	0.3333	0	0.1176	0.1143	0	0	0	0	1	1	1	0.8571	0.2692	0	0.9773
BM106B	2	G	1	0.9667	0.9667	1	0.6667	1	0.8824	0.8857	1	1	1	1	0	0	0	0.1429	0.7308	1	0.0227
BM10B	1	A	1	1	1	1	1	1	1	1	1	1	1	1	1	1	1	1	1	1	0.1923
BM10B	2	C	0	0	0	0	0	0	0	0	0	0	0	0	0	0	0	0	0	0	0.8077
BM113A	1	A	1	1	1	1	1	1	1	1	1	1	1	1	1	1	1	1	1	1	0
BM113A	2	T	0	0	0	0	0	0	0	0	0	0	0	0	0	0	0	0	0	0	1
BM118A	1	A	0	0	0	0	0.1042	0	0	0	0	0	0	0	0	0.0577	0.0926	0.2586	0	0	0.0357
BM118A	2	G	1	1	1	1	0.8958	1	1	1	1	1	1	1	1	0.9423	0.9074	0.7414	1	1	0.9643
BM11A	1	A	1	1	1	1	1	1	1	1	1	1	1	1	0.9833	1	1	1	1	1	0
BM11A	2	G	0	0	0	0	0	0	0	0	0	0	0	0	0.0167	0	0	0	0	0	1
BM12A	1	C	0	0	0	0	0.3519	0	0	0.0147	0	0	0	0	0.8167	0.9815	0.9828	1	1	0.9833	0.125
BM12A	2	T	1	1	1	1	0.6481	1	1	0.9853	1	1	1	1	0.1833	0.0185	0.0172	0	0	0.0167	0.875
BM12C	1	C	0.9483	1	0.9833	1	0.8654	1	0.9677	0.9839	0.9028	0.9655	1	0.9211	0.2143	0.0926	0.1897	0.7414	0.2963	0	0.6154
BM12C	2	T	0.0517	0	0.0167	0	0.1346	0	0.0323	0.0161	0.0972	0.0345	0	0.0789	0.7857	0.9074	0.8103	0.2586	0.7037	1	0.3846
BM147A	1	C	0.9667	0.9833	0.9667	0.8621	0.9259	0.9474	0.8529	0.9	0.9306	0.6897	0.4667	0.7273	1	0.9	0.931	0.7241	0.3148	0.2	0
BM147A	2	T	0.0333	0.0167	0.0333	0.1379	0.0741	0.0526	0.1471	0.1	0.0694	0.3103	0.5333	0.2727	0	0.1	0.069	0.2759	0.6852	0.8	1
BM151A	1	G	0.9483	0.931	0.9038	0.8966	0.9615	0.8611	0.9853	0.9857	0.9028	0	0	0	1	1	1	1	0	0	1
BM151A	2	T	0.0517	0.069	0.0962	0.1034	0.0385	0.1389	0.0147	0.0143	0.0972	1	1	1	0	0	0	0	1	1	0
BM16B	1	A	0.9	0.8793	0.8667	0.8621	0.7037	0.8889	0.803	0.8143	0.8611	0.7759	0.7333	0.803	0.8333	0.75	0.7931	0.7931	0.9074	0.95	0.9107
BM16B	2	G	0.1	0.1207	0.1333	0.1379	0.2963	0.1111	0.197	0.1857	0.1389	0.2241	0.2667	0.197	0.1667	0.25	0.2069	0.2069	0.0926	0.05	0.0893
BM17B	1	A	1	1	1	0.931	0.48	1	0.9853	1	0.9861	0.7586	0.5333	0.697	0.8667	0.6852	0	0	0.0185	0	0.1
BM17B	2	G	0	0	0	0.069	0.52	0	0.0147	0	0.0139	0.2414	0.4667	0.303	0.1333	0.3148	1	1	0.9815	1	0.9
BM201B	1	A	0	0	0	0	0	0	0	0	0	0	0	0	0	0	0	0	0	0	0.7115
BM201B	2	C	1	1	1	1	1	1	1	1	1	1	1	1	1	1	1	1	1	1	0.2885
BM201C	1	G	0	0	0	0	0.38	0	0	0	0	0	0.0167	0	0.0167	0.0577	0.5893	0.8571	0.4231	0.3276	0
BM201C	2	T	1	1	1	1	0.62	1	1	1	1	1	0.9833	1	0.9833	0.9423	0.4107	0.1429	0.5769	0.6724	1
BM202A	1	A	1	1	1	1	1	1	1	1	1	1	1	1	1	1	1	1	1	1	0
BM202A	2	C	0	0	0	0	0	0	0	0	0	0	0	0	0	0	0	0	0	0	1
BM202B	1	A	0	0	0	0	0	0	0	0	0	0	0	0	0	0	0	0	0	0	1
BM202B	2	T	1	1	1	1	1	1	1	1	1	1	1	1	1	1	1	1	1	1	0
BM203B	1	C	1	1	1	1	0.62	1	1	1	1	1	0.9833	1	0.9833	0.9259	0.4	0.1852	0.6087	0.6607	1
BM203B	2	T	0	0	0	0	0.38	0	0	0	0	0	0.0167	0	0.0167	0.0741	0.6	0.8148	0.3913	0.3393	0
BM203C	1	A	0.85	0.9138	0.9828	0.875	0.3913	0.9722	0.9091	0.8235	0.9697	0.2222	0.0333	0.0806	0.6724	0.4286	0.1296	0.0172	0.1111	0.0517	0
BM203C	2	G	0.15	0.0862	0.0172	0.125	0.6087	0.0278	0.0909	0.1765	0.0303	0.7778	0.9667	0.9194	0.3276	0.5714	0.8704	0.9828	0.8889	0.9483	1
BM203D	1	A	1	1	1	1	1	1	1	1	1	1	1	1	1	1	1	1	1	1	0
BM203D	2	T	0	0	0	0	0	0	0	0	0	0	0	0	0	0	0	0	0	0	1
BM204A	1	C	0	0	0	0	0.2963	0	0	0	0	0	0	0	0.0833	0.0556	0.3571	0.6429	0.2222	0.15	0
BM204A	2	T	1	1	1	1	0.7037	1	1	1	1	1	1	1	0.9167	0.9444	0.6429	0.3571	0.7778	0.85	1
BM21B	1	C	0.9833	0.9464	1	0.9464	0.5192	0.9737	1	0.9857	0.9583	0.6207	0.4167	0.4091	0.95	0	0	0	0.0185	0	0.9821
BM21B	2	G	0.0167	0.0536	0	0.0536	0.4808	0.0263	0	0.0143	0.0417	0.3793	0.5833	0.5909	0.05	1	1	1	0.9815	1	0.0179
BM21C	1	A	1	1	1	1	0.8148	1	1	1	1	1	1	1	0.95	0.0192	0.931	0.8966	0.963	0.9833	0.9821
BM21C	2	C	0	0	0	0	0.0185	0	0	0	0	0	0	0	0.05	0.9615	0.0172	0	0	0	0.0179
BM21C	3	T	0	0	0	0	0.1667	0	0	0	0	0	0	0	0	0.0192	0.0517	0.1034	0.037	0.0167	0
BM26B	1	A	1	1	1	1	0.963	1	1	1	1	1	1	1	1	1	0.9483	0.8448	0.963	1	0
BM26B	2	T	0	0	0	0	0.037	0	0	0	0	0	0	0	0	0	0.0517	0.1552	0.037	0	1
BM2G	1	G	1	1	1	1	1	1	1	1	1	1	1	1	1	1	1	1	1	1	0.0556
BM2G	2	T	0	0	0	0	0	0	0	0	0	0	0	0	0	0	0	0	0	0	0.9444
BM30A	1	A	1	1	1	1	1	1	1	1	1	1	1	1	1	1	1	1	1	1	0.1429
BM30A	2	G	0	0	0	0	0	0	0	0	0	0	0	0	0	0	0	0	0	0	0.8571
BM30C	1	A	0	0	0	0	0	0	0	0	0	0	0	0	0	0	0	0	0	0	0.5179
BM30C	2	T	1	1	1	1	1	1	1	1	1	1	1	1	1	1	1	1	1	1	0.4821
BM32A	1	A	0.4138	0.3621	0.431	0.4138	0.48	0.3947	0.4559	0.4853	0.6029	0.3393	0.3667	0.2759	0.3333	0.5556	0.375	0.5385	0.7778	0.9038	0.1296
BM32A	2	G	0.5862	0.6379	0.569	0.5862	0.52	0.6053	0.5441	0.5147	0.3971	0.6607	0.6333	0.7241	0.6667	0.4444	0.625	0.4615	0.2222	0.0962	0.8704
BM33B	1	A	1	1	1	1	1	1	1	1	1	1	1	1	1	1	1	1	0.963	0.9821	0.5417
BM33B	2	T	0	0	0	0	0	0	0	0	0	0	0	0	0	0	0	0	0.037	0.0179	0.4583
BM35C	1	A	0.8333	0.931	0.8667	0.9138	0.5556	0.9474	0.7941	0.8824	0.9143	0.4107	0.3	0.3594	0.4833	0.56	0.3889	0.2759	0.0769	0.0667	0.9286
BM35C	2	T	0.1667	0.069	0.1333	0.0862	0.4444	0.0526	0.2059	0.1176	0.0857	0.5893	0.7	0.6406	0.5167	0.44	0.6111	0.7241	0.9231	0.9333	0.0714
BM36F	1	A	1	1	1	1	1	1	1	1	1	1	1	1	0.9483	0.9167	0.9655	0.9107	0.9792	1	0.0179
BM36F	2	C	0	0	0	0	0	0	0	0	0	0	0	0	0.0517	0.0833	0.0345	0.0893	0.0208	0	0.9821
BM38B	1	A	0.1833	0.2069	0.15	0.1724	0.2037	0.0789	0.2188	0.2424	0.2639	0.1034	0.1552	0.0909	0.0333	0.0741	0.1552	0.2759	0.463	0.9333	0.7143
BM38B	2	G	0.8167	0.7931	0.85	0.8276	0.7963	0.9211	0.7812	0.7576	0.7361	0.8966	0.8448	0.9091	0.9667	0.9259	0.8448	0.7241	0.537	0.0667	0.2857
BM44B	1	A	0	0	0	0	0	0	0	0	0	0	0	0	0	0	0	0	0	0	0.537
BM44B	2	G	1	1	1	1	1	1	1	1	1	1	1	1	1	1	1	1	1	1	0.463
BM50B	1	A	0	0	0	0	0	0	0	0	0	0	0	0	0	0	0	0	0	0	0.0714
BM50B	2	G	1	1	1	1	1	1	1	1	1	1	1	1	1	1	1	1	1	1	0.9286
BM54A	1	A	1	1	1	1	1	1	1	1	1	1	1	1	1	1	1	1	1	1	0
BM54A	2	G	0	0	0	0	0	0	0	0	0	0	0	0	0	0	0	0	0	0	1
BM57A	1	C	1	0.9833	0.9833	1	0.5926	1	0.9853	1	0.9306	0.3966	0.1607	0.3333	1	1	0.4138	0.3462	0.8889	1	1
BM57A	2	T	0	0.0167	0.0167	0	0.4074	0	0.0147	0	0.0694	0.6034	0.8393	0.6667	0	0	0.5862	0.6538	0.1111	0	0
BM57D	1	A	0	0	0	0	0.3654	0	0	0	0	0	0	0	0	0	0.5	0.6071	0.1154	0	0
BM57D	2	C	1	1	1	1	0.6346	1	1	1	1	1	1	1	1	1	0.5	0.3929	0.8846	1	1
BM5B	1	A	1	1	1	1	0.9074	1	1	1	1	1	1	1	1	1	1	0.8966	1	1	0.9286
BM5B	2	G	0	0	0	0	0.0926	0	0	0	0	0	0	0	0	0	0	0.1034	0	0	0.0714
BM5D	1	C	1	1	1	1	0.8519	1	1	1	1	1	0.9667	1	0.8	0.4259	0.8103	0.8621	0.9815	1	0.8036
BM5D	2	T	0	0	0	0	0.1481	0	0	0	0	0	0.0333	0	0.2	0.5741	0.1897	0.1379	0.0185	0	0.1964
BM61A	1	C	0.3393	0.3462	0.3036	0.2885	0.6852	0.4474	0.2097	0.4375	0.3194	0.8929	0.8913	0.9286	0.4	0.6667	0.8036	0.9259	0.2105	0	0.1786
BM61A	2	T	0.6607	0.6538	0.6964	0.7115	0.3148	0.5526	0.7903	0.5625	0.6806	0.1071	0.1087	0.0714	0.6	0.3333	0.1964	0.0741	0.7895	1	0.8214
BM62A	1	A	0	0.0167	0	0	0	0	0	0	0.0278	0.0345	0.1833	0.2273	0.05	0	0	0	0	0	1
BM62A	2	G	1	0.9833	1	1	1	1	1	1	0.9722	0.9655	0.8167	0.7727	0.95	1	1	1	1	1	0
BM64A	1	C	1	1	1	1	1	1	1	1	1	1	1	1	1	1	1	1	1	1	0
BM64A	2	T	0	0	0	0	0	0	0	0	0	0	0	0	0	0	0	0	0	0	1
BM67C	1	A	0.5167	0.65	0.55	0.4655	0.3519	0.5833	0.5	0.5441	0.5972	0.0517	0.1333	0.0303	0.7414	0.6923	0.6034	0.0345	0.037	0	0.0536
BM67C	2	T	0.4833	0.35	0.45	0.5345	0.6481	0.4167	0.5	0.4559	0.4028	0.9483	0.8667	0.9697	0.2586	0.3077	0.3966	0.9655	0.963	1	0.9464
BM6C	1	C	0.9286	0.8966	0.9	0.4643	0.1667	0.8684	0.6818	0.8857	0.8472	1	0.9833	1	0.3667	0.0926	0	0	0	0	0.4286
BM6C	2	T	0.0714	0.1034	0.1	0.5357	0.8333	0.1316	0.3182	0.1143	0.1528	0	0.0167	0	0.6333	0.9074	1	1	1	1	0.5714
BM78B	1	A	0.1731	0.0833	0.0741	0.1739	0.34	0.0312	0.05	0.069	0.0484	0	0	0	0	0.1667	0.5179	0.7931	0.9815	0.9833	0.037
BM78B	2	G	0.8269	0.9167	0.9259	0.8261	0.66	0.9688	0.95	0.931	0.9516	1	1	1	1	0.8333	0.4821	0.2069	0.0185	0.0167	0.963
BM8E	1	A	0	0	0	0	0	0	0	0	0	0	0	0	0.0167	0.0185	0	0	0	0	1
BM8E	2	G	1	1	1	1	1	1	1	1	1	1	1	1	0.9833	0.9815	1	1	1	1	0
BM92B	1	A	1	1	1	1	1	1	1	1	1	1	1	1	1	1	1	1	1	1	0
BM92B	2	T	0	0	0	0	0	0	0	0	0	0	0	0	0	0	0	0	0	0	1
BM9B	1	A	0.4828	0.5714	0.5833	0.4107	0.2407	0.5294	0.4853	0.4714	0.5278	0.5862	0.65	0.5758	0.6481	0.5455	0.3276	0.1429	0.0185	0.0167	0.3
BM9B	2	G	0.5172	0.4286	0.4167	0.5893	0.7593	0.4706	0.5147	0.5286	0.4722	0.4138	0.35	0.4242	0.3519	0.4545	0.6724	0.8571	0.9815	0.9833	0.7
BM9C	1	A	0.3929	0.375	0.4138	0.5357	0.7593	0.4474	0.5147	0.5147	0.4583	0.2759	0.3	0.3636	0.2414	0.3077	0.6724	0.8704	0.9815	0.9833	0.0357
BM9C	2	C	0.0357	0.0536	0.069	0.0536	0.0556	0.0789	0.0588	0.0441	0.0833	0.0172	0	0.0152	0.1207	0.0769	0	0	0	0	0
BM9C	3	T	0.5714	0.5714	0.5172	0.4107	0.1852	0.4737	0.4265	0.4412	0.4583	0.7069	0.7	0.6212	0.6379	0.6154	0.3276	0.1296	0.0185	0.0167	0.9643
