# Supplementary figures and images for: Single nucleotide polymorphisms in native South American Atlantic coast populations of smooth shelled mussels: hybridization with invasive European Mytilus galloprovincialis
Source: Genet Sel Evol. 2018 Feb 22;50:5. doi: 10.1186/s12711-018-0376-z (PMC5824471; doi:10.1186/s12711-018-0376-z)

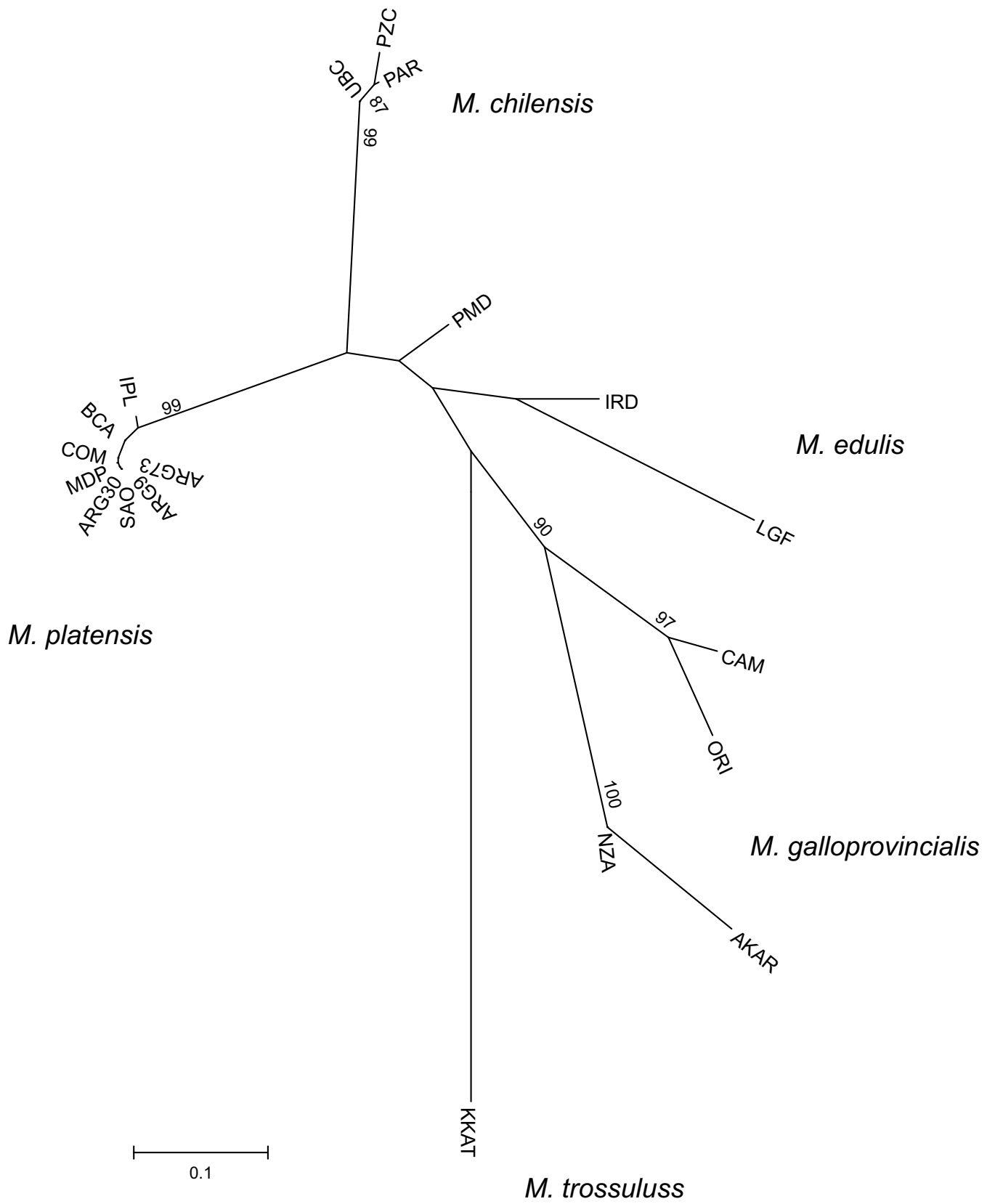

Supplement: Supplementary file 3 — Additional file 3: Figure S1. Neighbour-joining tree of native South American and the reference Mytilus taxa. Description: Neighbour-joining tree shows genetic relationship between 19 Mytilus spp. samples from Argentina and reference populations of M. edulis, M. trossulus, M. galloprovincialis and M. chilensis from America, Europe and New Zealand based on the FST distance measures obtained with POPTREEW and visualised with MEGA version 6. [file 12711_2018_376_MOESM3_ESM.pdf]
