# Supplementary material for: Single nucleotide polymorphisms in native South American Atlantic coast populations of smooth shelled mussels: hybridization with invasive European Mytilus galloprovincialis
Source: Genet Sel Evol. 2018 Feb 22;50:5. doi: 10.1186/s12711-018-0376-z (PMC5824471; doi:10.1186/s12711-018-0376-z)
Supplement: Supplementary file 4 — Additional file 4: Table S3. FST distance matrix for 19 Mytilus spp. samples for 51 SNPs. Description: Values of FST with P < 0.05 after Benjamini–Yekutieli (FDR-BY) correction are marked in bold. Site names and locations are in Table 1. [file 12711_2018_376_MOESM4_ESM.pdf]

Table S3. F_{ST} distance matrix for 51 SNP.

name	ARG30	ARG73	ARG9	IPL	PMD	SAO	BCA	COM	MDP	UBC	PZC	PAR	IRD	LGF	CAM	ORI	NZA	AKAR
ARG73	-0.005																	
ARG9	-0.007	-0.007																
IPL	**0.043**	**0.051**	**0.049**															
PMD	**0.290**	**0.299**	**0.307**	**0.228**														
SAO	-0.004	-0.009	-0.011	**0.036**	**0.273**													
BCA	0.013	0.025	0.016	0.010	**0.260**	0.024												
COM	-0.001	0.007	0.009	**0.042**	**0.271**	0.006	0.009											
MDP	0.005	0.006	0.003	**0.039**	**0.290**	0.006	0.012	0.005										
UBC	**0.349**	**0.372**	**0.361**	**0.357**	**0.285**	**0.351**	**0.365**	**0.350**	**0.364**									
PZC	**0.439**	**0.456**	**0.450**	**0.435**	**0.297**	**0.441**	**0.443**	**0.436**	**0.445**	**0.041**								
PAR	**0.401**	**0.422**	**0.415**	**0.403**	**0.290**	**0.405**	**0.413**	**0.401**	**0.410**	0.007	**0.028**							
IRD	**0.356**	**0.365**	**0.363**	**0.347**	**0.279**	**0.365**	**0.319**	**0.334**	**0.362**	**0.459**	**0.494**	**0.475**						
LGF	**0.555**	**0.561**	**0.566**	**0.523**	**0.319**	**0.557**	**0.521**	**0.531**	**0.546**	**0.549**	**0.552**	**0.543**	**0.261**					
CAM	**0.606**	**0.611**	**0.616**	**0.570**	**0.208**	**0.597**	**0.583**	**0.591**	**0.603**	**0.544**	**0.515**	**0.526**	**0.419**	**0.368**				
ORI	**0.650**	**0.659**	**0.661**	**0.611**	**0.217**	**0.644**	**0.626**	**0.633**	**0.646**	**0.565**	**0.534**	**0.553**	**0.533**	**0.490**	**0.118**			
NZA	**0.661**	**0.676**	**0.671**	**0.610**	**0.307**	**0.667**	**0.625**	**0.643**	**0.644**	**0.544**	**0.505**	**0.519**	**0.554**	**0.522**	**0.348**	**0.338**		
AKAR	**0.769**	**0.784**	**0.778**	**0.729**	**0.475**	**0.796**	**0.730**	**0.746**	**0.743**	**0.674**	**0.636**	**0.649**	**0.677**	**0.661**	**0.538**	**0.550**	**0.113**	
KKAT	**0.797**	**0.803**	**0.801**	**0.784**	**0.705**	**0.795**	**0.784**	**0.790**	**0.793**	**0.776**	**0.769**	**0.777**	**0.755**	**0.776**	**0.765**	**0.778**	**0.796**	**0.847**

Values with P<0.05 after Benjamini–Yekutieli (FDR-BY) correction is marked in bold. See Table 1 for site name definition.
