# Supplementary material for: Single nucleotide polymorphisms in native South American Atlantic coast populations of smooth shelled mussels: hybridization with invasive European Mytilus galloprovincialis
Source: Genet Sel Evol. 2018 Feb 22;50:5. doi: 10.1186/s12711-018-0376-z (PMC5824471; doi:10.1186/s12711-018-0376-z)
Supplement: Supplementary file 5 — Additional file 5: Table S4. Result of population assignment algorithms STRUCTURE and GeneClass for 19 populations of mussels. Description: Two methods of analysis were used to test the assignment of individuals from Argentina to the most likely population and regions, based on reference taxa. In the STRUCTURE analysis at K = 5, most individuals were properly assigned to their original samples. Individuals were assigned with GeneClass2 to baseline populations based on region of origin with a success rate of 97.8%. [file 12711_2018_376_MOESM5_ESM.pdf]

Table S4. Result of population assignment algorithms STRUCTURE and GeneClass for 19 populations of mussels.

	STRUCTURE (K=5)					GeneClass		Assigned to species				GeneClass		Assigned of individuals to origin region						
			Clusters																	
	1	2	3	4	5															
Name	*M. platensis*	*M. chilensis*	*M. edulis*	*M. galloprovincialis*	*M. trossulus*	*M. platensis*	*M. chilensis*	*M. edulis*	*M. galloprovincialis*	*M. trossulus*	ARG30, ARG73, ARG9, IPL, SAO, BCA, COM, MDP	PMD	UBC, PZC, PAR	IRD	LGF	CAM	ORI	NZA	AKAR	KKAT
ARG30	30 (100%)					30 (100%)					30 (100%)									
ARG73	29 (96.67%)	1 (3.33%)				29 (96.67%)	1 (3.33%)				29 (96.67%)		1 (3.33%)							
ARG9	30 (100%)					30 (100%)					30 (100%)									
IPL	29 (100%)					29 (100%)					29 (100%)									
PMD	8 (29.63%)	1 (3.7%)	1 (3.7%)	17 (62.96%)		10 (37.04%)	1 (3.7%)	4 (14.81%)	12 (44.44%)		2 (7.4%)	24 (88.9%)					1 (3.7%)			
SAO	19 (100%)					19 (100%)					19 (100%)									
BCA	34 (100%)					34 (100%)					34 (100%)									
COM	35 (100%)					35 (100%)					35 (100%)									
MDP	36 (100%)					36 (100%)					36 (100%)									
UBC	1 (3.45%)	28 (96.55%)				1 (3.45%)	28 (96.55%)				1 (3.45%)		28 (96.55%)							
PZC		30 (100%)					30 (100%)						30 (100%)							
PAR		33 (100%)					33 (100%)						33 (100%)							
IRD	1 (3.33%)		29 (96.67%)			1 (3.33%)		29 (96.67%)			1 (3.33%)			29 (96.67%)						
LGF			27 (100%)					27 (100%)							27 (100%)					
CAM				29 (100%)					29 (100%)							26 (89.65%)	3 (10.35%)			
ORI				29 (100%)					29 (100%)							1 (3.45%)	28 (96.55%)			
NZA				27 (100%)					27 (100%)									24 (88,9%)	3 (11.1%)	
AKAR				30 (100%)					30 (100%)										30 (100%)	
KKAT					28 (100%)					28 (100%)										28 (100%)

GeneClass

Assigned PMD individuals to study populations after removal PMD sample from baseline populations

IPL	ARG30	PZC	IRD	LGF	CAM	ORI
9(33.33)	1 (3.7%)	1 (3.7%)	4(14.81%)		5(18.52)	7(25.93)
