# Supplementary material for: Single nucleotide polymorphisms in native South American Atlantic coast populations of smooth shelled mussels: hybridization with invasive European Mytilus galloprovincialis
Source: Genet Sel Evol. 2018 Feb 22;50:5. doi: 10.1186/s12711-018-0376-z (PMC5824471; doi:10.1186/s12711-018-0376-z)
Supplement: Supplementary file 6 — Additional file 6: Table S5. FST distance matrix for Mytilus spp. samples. Description: FST distance matrix is presented for Mytilus spp. samples from Argentina and reference populations of M. edulis, M. trossulus, M. galloprovincialis and M. chilensis, obtained with POPTREEW for individuals without admixture (q > 0.8 or q < 0.2) identified by STRUCTURE analysis. [file 12711_2018_376_MOESM6_ESM.pdf]

Table S5. FST distance matrix for 51 SNP.

	ARG9	SAO	UBC	PAR	PZC	LGF	IRD	CAM	ORI	NZA	AKAR	KKAT
ARG30	-0.005	0.001	0.359	0.398	0.443	0.531	0.395	0.601	0.639	0.66	0.764	0.781
ARG9		-0.01	0.387	0.428	0.468	0.547	0.409	0.62	0.657	0.682	0.785	0.79
SAO			0.393	0.433	0.477	0.552	0.423	0.624	0.662	0.69	0.796	0.795
UBC				0.003	0.032	0.557	0.49	0.543	0.568	0.567	0.689	0.768
PAR					0.028	0.559	0.508	0.533	0.56	0.559	0.685	0.768
PZC						0.571	0.523	0.525	0.549	0.548	0.67	0.765
LGF							0.25	0.411	0.526	0.57	0.685	0.77
IRD								0.442	0.552	0.593	0.703	0.754
CAM									0.097	0.343	0.52	0.755
ORI										0.353	0.539	0.766
NZA											0.103	0.791
AKAR												0.84

Values with P<0.05 after Benjamini–Yekutieli (FDR-BY) correction is marked in bold. See Table 1 for site name definition.
